# Human umbilical cord mesenchymal stem cells derived extracellular vesicles alleviate salpingitis by promoting M1–to–M2 transformation

**DOI:** 10.3389/fphys.2023.1131701

**Published:** 2023-02-16

**Authors:** Changlin Zhang, Wei Liao, Weizhao Li, Mengxiong Li, Xiaoyu Xu, Haohui Sun, Yaohua Xue, Lixiang Liu, Jiehong Qiu, Chi Zhang, Xunzhi Zhang, Juntong Ye, Jingran Du, David Y. B. Deng, Wuguo Deng, Tian Li

**Affiliations:** ^1^ Pelvic Floor Disorders Center, Scientific Research Center, Department of Gynecology, The Seventh Affiliated Hospital of Sun Yat-sen University, Shenzhen, China; ^2^ Sun Yat-Sen University Cancer Center, State Key Laboratory of Oncology in South China, Collaborative Innovation Center of Cancer Medicine, Guangzhou, China; ^3^ Department of Clinical Laboratory, Dermatology Hospital, Southern Medical University, Guangzhou, China; ^4^ College of Life Sciences and Oceanography, Shenzhen University, Shenzhen, China

**Keywords:** chronic salpingitis, macrophage polarization, NF-κB signaling pathway, mesenchymal stem cell (MSC), extracellular vesicles

## Abstract

**Background:** With an increasing number of patients experiencing infertility due to chronic salpingitis after *Chlamydia trachomatis* (CT) infection, there is an unmet need for tissue repair or regeneration therapies. Treatment with human umbilical cord mesenchymal stem cell-derived extracellular vesicles (hucMSC-EV) provides an attractive cell-free therapeutic approach.

**Methods:** In this study, we investigated the alleviating effect of hucMSC-EV on tubal inflammatory infertility caused by CT using *in vivo* animal experiments. Furthermore, we examined the effect of hucMSC-EV on inducing macrophage polarization to explore the molecular mechanism.

**Results:** Our results showed that tubal inflammatory infertility caused by *Chlamydia* infection was significantly alleviated in the hucMSC-EV treatment group compared with the control group. Further mechanistic experiments showed that the application of hucMSC-EV induced macrophage polarization from the M1 to the M2 type *via* the NF-κB signaling pathway, improved the local inflammatory microenvironment of fallopian tubes and inhibited tube inflammation.

**Conclusion:** We conclude that this approach represents a promising cell-free avenue to ameliorate infertility due to chronic salpingitis.

## Graphical Abstract

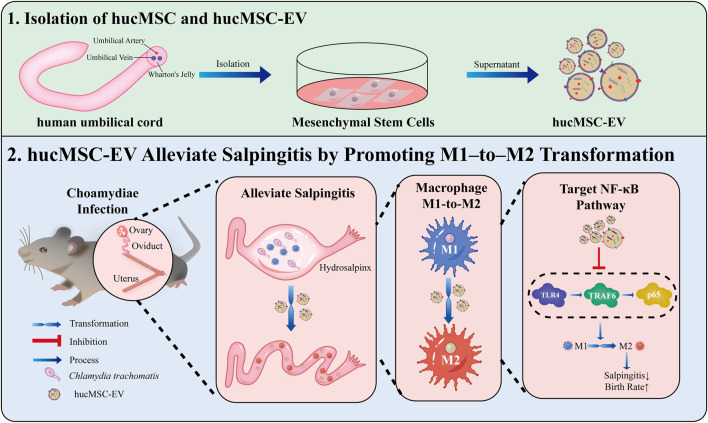

## 1 Introduction

Having a healthy and happy baby is vital to the wellbeing of millions of families; however, ∼15% of couples worldwide (∼50 million couples) experience infertility (Tsevat et al., 2017). In 2013, the World Health Organization (WHO) estimated that 25% of couples in developing countries could not meet their fertility needs, with female infertility accounting for ∼50% of infertility cases (Newman et al., 2015). Tubal factor (fallopian tubes) infertility accounts for ∼10%–30% of female infertility (Tsevat et al., 2017).

*Chlamydia trachomatis* (CT) infection is the main cause of fallopian tube obstruction and infertility, responsible for ∼43% of cases (Ruijin Shao et al., 2012). The infection persists in many patients who received treatment, causing pathological damage and ultimately leading to fibrosis and scar formation, damage to the normal function of fallopian tubes, and, in serious cases, fallopian tube obstruction and infertility (Haggerty et al., 2010).

The mechanism of fallopian tube injury caused by CT infection remains unclear and may be related to ontogenetic factors, cytokine activation, immune escape, and co-infection (Brunham, 2022). The formation of fallopian tube scars is not only due to the destruction of the fallopian tube epithelial cells by CT but also due to the accumulation of inflammatory cells, release of cytokines, and activation of the complement system in the immune microenvironment (Murthy et al., 2018). Macrophages play a crucial role in the occurrence and development of inflammatory diseases and are classified into two main types: M1, generated by classical activation, and M2, generated by selective activation (Shapouri-Moghaddam et al., 2018; Galipeau, 2021; Pouyanfard et al., 2021).

Mesenchymal stem cells (MSCs) are adult stem cells derived from mesoderm with high self-renewal ability and multidirectional differentiation potential (Dominici et al., 2006; Andrzejewska et al., 2019).

At present, MSCs are rarely used in the treatment of tubal factor infertility, especially tubal infertility. Tubal infertility is closely related to inflammatory adhesions (Ling et al., 2022). The formation of inflammatory adhesions is a mechanism that protects the body against external stimuli (Medzhitov, 2008). It can limit inflammation, which is conducive to the repair of tissue damage (Cooke, 2019; Yuan et al., 2021; Li-Tao Shao et al., 2022); however, under certain conditions, it can cause adhesion and infertility (Hafner, 2015). MSCs exert their functions through the paracrine pathway. *In vitro* and *in vivo* studies have confirmed that the culture supernatant of MSCs can inhibit the abnormal activation of T cells after co-culture *in vitro* (Negi and Griffin, 2020; Markov et al., 2021). Injections of the culture supernatant of MSCs into a mouse model of renal injury effectively reduced the area of injured tissue (Erpicum et al., 2017). This suggests that factors in the culture supernatant of MSCs exert functions similar to those of MSCs themselves.

Treatments based on umbilical cord derived mesenchymal stem cells (MSCs) have shown some promising achievements (V Yeung et al., 2019). Our previous study showed that MSCs transplantation can markedly reduce inflammation in salpingitis caused by CT infection (Liao et al., 2019). However, their potential tumorigenicity (Webber et al., 2015), low perfusion, low retention and other limitations are still controversial, which limit the clinical application of MSCs. In contrast, hucMSC-EV exhibit a similar function to their source cells and are expected to overcome these limitations. EV are tiny vesicles (40–100 nm in diameter) secreted out of cells by membrane fusion (Pegtel and Gould, 2019). EV contain active substances, such as nucleic acids and proteins, which are transferred to target tissues to perform their functions (Simons and Raposo, 2009; Pluchino and Smith, 2019). MSC-extracellular vesicles (MSC-EV) participate in tissue-damage repair (Yin et al., 2019; Vincent Yeung et al., 2022), immune regulation (Harrell et al., 2019; Farhat et al., 2022), and reshaping of the immune microenvironment (Seo et al., 2019). In addition, compared with MSC treatment, exosome components are relatively simple, and their structural characteristics enable them to reach the target tissues with high efficiency, likely avoiding the risk of long-term abnormal differentiation and tumor formation caused by stem-cell transplantation in the host (Vizoso et al., 2017; Ning et al., 2018).

Our previous study showed that MSC transplantation can markedly reduce inflammation in salpingitis caused by CT infection (Liao et al., 2019). The role of MSCs in the repair and reconstruction of inflammatory adhesions may be related to the regulation of different inflammatory environments and inflammatory cells (Shi et al., 2018). *In vitro* studies have confirmed that MSCs can promote the transformation of macrophages into the M2 type through the paracrine pathway, suggesting that MSC-induced M2 macrophages are important components in the treatment of oviduct inflammatory injury (Liao et al., 2019). Based on the results of our previous *in vivo* and *in vitro* studies and literature reports, we propose the following hypothesis: MSCs regulate macrophage polarization to the M2 type by secreting extracellular vesicles and change the level of cytokines secreted by macrophages, reducing local inflammatory responses in the oviduct and promoting tissue-damage repair.

The aim of this study was to use hucMSC-EV to treat CT salpingitis and explore the relationship between hucMSC-EV and macrophage polarization *in vitro* and *in vivo*, as well as the regulation of cytokine levels secreted by macrophages, to provide new ideas for the treatment of salpingitis caused by CT infection.

## 2 Methods

### 2.1 *In vitro* experiments

We collected fresh umbilical cords from full-term, cesarean section puerperae. The puerperae had tested negative for gestational diabetes mellitus, infection, fever, and autoimmune or other diseases. The acquisition of the human umbilical cords (hucs) was approved by the Ethics Committee of the Seventh Affiliated Hospital, Sun Yat-sen University (KY-2022-007-01). The puerperae were fully informed and consented.

The fresh umbilical cords were processed in a biosafety cabinet within 6 h. First, the cords were rinsed twice with 1×PBS containing 100 U/mL penicillin and 100 mg/mL streptomycin (BL505A; Biosharp, Hefei, China). Arteries, veins, blood vessels, and epithelial cells were removed under aseptic conditions to obtain Wharton’s jelly, which was cut into 1 mm^3^ pieces and placed at the bottom of T25 culture flasks. The flasks were incubated at 37°C with 5% CO_2_ for 30 min. Next, 1.5 mL DMEM/F12 medium containing 15% FBS (164210; Procell, Wuhan, China), 100 U/mL penicillin, and 100 mg/mL streptomycin was added to fully soak the pieces. The flasks were placed in a 37°C incubator with 5% CO_2_, and the medium was gently replaced every 3 days. Approximately 10 days later, fibroblast-like MSCs were observed around the umbilical cord pieces. The umbilical cord pieces were removed, and the cells were marked as passage zero.

To investigate the capacity of the isolated MSCs to adhere to plastic in standard culture conditions, we used an inverted phase-contrast microscope (ECLIPSE C1; Nikon, Japan). MSCs were identified using flow-cytometric analysis (FACSCalibur™; Becton Dickinson) to detect cell surface markers (CD19, CD34, CD45, CD73, CD90, CD105, and HLA-DR). When 90%–100% confluency was reached, we used trypsin to prepare cell suspensions at a concentration of 1 × 10^6 cells/mL. Cells were incubated with antibodies on ice under dark conditions for 30 min. Cells were washed thrice with 1 × PBS to remove the uncombined antibodies and analyzed using a flow cytometer within 1 h.

### 2.2 Differentiation of HucMSCs

To evaluate differentiation capacity, MSCs were cultured with osteogenic (PD-003; Procell, Wuhan, China) or adipogenic media (PD-004; Procell, Wuhan, China) according to protocols. The medium was replaced with a fresh differentiation medium every 3 days. After 3 weeks, the medium was removed, and the cells were washed thrice with 1 × PBS, fixed with 4% neutral formalin for 15 min, and washed thrice with 1 × PBS. The osteogenic and adipogenic cells were stained with alizarin red S (ARS) and oil red O, respectively, for 30 min at 22–25°C and washed thrice with PBS, and observed under a microscope.

### 2.3 Extraction of hucMSC-EV

We used ultracentrifugation to collect extracellular vesicles from the culture supernatant of hucMSCs (3rd–5th passage cells). When cell density reached 70%–80%, we removed the medium, washed the cells thrice with 1×PBS, and added serum-free DMEM/F12 for 48 h to exclude the influence of EV from fetal blood serum. We collected the culture supernatant and collected EV immediately or froze them at −80°C. The culture supernatant was centrifuged at 10 000 × *g* for 45 min at 4°C to remove unwanted cells and cell debris. To obtain higher-purity EV, the collected supernatant was filtered through a 0.22-μm filter (SLGP033RB-0.22; Merck Millipore, United States) and ultracentrifuged (JXN-30; Beckman, United States) at 108 000 × *g* for 70 min at 4°C (Optima L-90K; Beckman, United States). The supernatant was discarded, and the pellet was resuspended in 1×Dulbecco’s phosphate-buffered saline (DPBS) to remove unwanted proteins. The samples were ultracentrifuged at 108 000 × *g* for 70 min at 4°C and resuspended in 200 μL of 1×DPBS to obtain high-density, pure EV.

### 2.4 Identification of hucMSC-EV

#### 2.4.1 Electron microscopy

Twenty microliters of extracellular vesicles suspension were transferred onto a copper grid with a carbon film for 3–5 min. A 2% phosphotungstic acid solution was added to the copper grid. After 1–2 min to allow for staining, excess liquid was blotted using filter paper, and the grid was left to dry at room temperature. The cuprum grids were observed under TEM (HT7800; HITACHI, Japan), and images were captured.

#### 2.4.2 Western blot

The adherent cells were scraped off the dish using trypsin or a plastic cell scraper, and the cell pellet was collected after centrifugation. To acquire cell lysates, RIPA buffer (P0013B; Beyotime, Shanghai, China) was added to the cell pellet on ice. After 30 min, the cell lysate was centrifuged at 4°C for 10 min to remove the dissolved pellet. A BCA kit (P0012; Beyotime, Shanghai, China) was used to quantify protein. After adding the appropriate loading buffer, each cell lysate was boiled at 100°C for 10 min. A 10% SDS-PAGE was prepared using a PAGE gel fast preparation kit (PG112; EpiZyme, Shanghai), and equal amounts of protein were loaded into the wells, along with a molecular weight marker (26616; Thermo Fisher, United States). The gel was run for 50 min at 150 V. Proteins were transferred to PVDF membranes (IPVH00010; Merck Millipore, United Statesa) and blocked for 1 h in 5% skim milk (LP0033B; Oxoid, United Kingdom) at room temperature. The membranes were incubated overnight at 4°C with primary antibodies from Proteintech (Rosemont, IL, United States) against CD81 (27855-1-AP, rabbit; 1:1000), Tsg101 (28283-1-AP, rabbit; 1:2000), calnexin (10427-2-AP, rabbit; 1:20000), P65 (10745-1-AP, rabbit; 1:1000), TLR4 (19811-1-AP, rabbit; 1:1000), glyceraldehyde-3-phosphate dehydrogenase (GAPDH, 10494-1-AP, rabbit; 1:1000), and tumor necrosis factor receptor-associated factor 6 (TRAF6) (#67591, rabbit; 1:1000) (Cell Signaling Technology, Beverly, MA, United States). The membranes were washed thrice with PBST (10 min per wash), re-probed with HRP-conjugated Affinipure Goat Anti-Rabbit IgG (SA00001-2; 1:10,000, Proteintech) at room temperature for 1 h, and washed thrice with PBST (10 min per wash). The membranes were covered with chemiluminescent HRP substrate (WBKLS0100; Merck Millipore, United States), and images were acquired using a ChemiDoc Touch Imaging System (1708370; Bio-Rad, United States). The gray value of the target protein bands was quantified using ImageJ 1.53k software, using GAPDH for normalization.

#### 2.4.3 Nanoparticle Tracking Analysis

To measure the particle size of purified hucMSC-EV, we chose Nanoparticle Tracking Analysis using NanoSight (NS300; Malvern, United Kingdom). The hucMSC-EV suspension (20 μL) was diluted to 1 mL using 1×DPBS, and the diluent was pumped into the NanoSight device avoiding air bubbles.

### 2.5 Animal experiments

#### 2.5.1 Establishment and treatment of the murine chronic salpingitis model

Four-week-old male and female C3H mice were obtained from VITAL RIVER (Beijing, China) and housed in an SPF barrier system in appropriate facilities (certificate number: SYXK [Guang-dong] 2015-0102), seeing the attached Supplementary Figure S1 for the flow chart of the experiment. All animal experiments were conducted according to the regulations of the Institutional Animal Care and Use Committee at Sun Yat-sen University Cancer Center (Certificate Number: KY-2022-007-01).

The 24 female C3H mice were subcutaneously injected with 2.5 mg of medroxyprogesterone at days 3 and 7. After 1 week, the female mice were transvaginally injected with 1 × 10^7^ inclusion-forming units MoPn *chlamydia* (ATCC VR-123TM; United States). To prevent *chlamydia* flow out of the vagina, the mice were hung upside down for 1 min after injection.

After 2 weeks, the female mice were randomly divided into three groups—hucMSC-EV, DPBS, and DMEM—of eight mice. The mice in the hucMSC-EV group were transvaginally injected with 25 μL of 100 μg/mL hucMSC-EV three times every 3 days.

After 2 weeks, three mice from each group were randomly selected to be sacrificed. The enterocoelia were exposed to observe the fallopian tubes and acquire images. The fallopian tubes were dissected in a 4% paraformaldehyde solution or liquid nitrogen for subsequent detection. To test the fertilization capacity of female mice, the remaining five female mice of each group were cohoused with two male mice in a cage for 2 weeks. When the vaginal plug was observed, the female mouse was considered fertilized; feeding continued for 7 days to determine the presence of embryos in the uterus, whereby, female mice were marked as pregnant.

#### 2.5.2 Histology and immunofluorescence

Fresh fallopian tubes were fixed in 4% paraformaldehyde (BL539A; Biosharp, Hefei) for >24 h, and then was dehydrated, paraffin-embedded, and cut into 4-μm-thick sections, The sections were deparaffinized, rehydrated using Xylene and alcohol gradients, and stained with Hematoxylin and Eosin (H&E). The stained sections were dehydrated and sealed with neutral gum. Observation, image acquisition, and analysis were performed under a microscope.

We used immunofluorescence to identify macrophages in the fallopian tubes. The slides were deparaffinized, rehydrated, and immersed in EDTA antigen retrieval buffer (pH 8.0). After blocking with serum, the slides were incubated with CD206 primary antibody (GB13438, Rabbit; Servicebio; 1:500), followed by CY3-conjugated Affinipure Goat Anti-Rabbit IgG secondary antibody (GB21303; Servicebio; 1:300). DAPI was used for nuclear staining. The slides were coverslipped using an antifade mounting medium. Images were obtained using fluorescent microscopy (ECLIPSE C1; Nikon, Japan).

### 2.6 RAW264.7 macrophage uptake of membrane components

RAW264.7 (Donated by Professor Deng Wuguo from Sun Yat-Sen University Cancer Center) cells were To verify that macrophage RAW264.7 cells could uptake membrane components of the hucMSC culture supernatant, the PKH67 Green Fluorescent Cell Linker Mini Kit (MINI67-1KT; Sigma, Germany) was used. Twenty microliters of PKH67 solution were mixed with 10 mL of hucMSC culture supernatant. RAW264.7 cells were plated in 6-well culture dishes; when 70%–80% confluency was reached, the cells were washed thrice with 1×PBS. For nuclear staining, DAPI (C0065-10; Solarbio, Beijing) was added to the wells for 10 min at room temperature. After washing thrice with 1×PBS, the PKH67 working solution was added; the plates were incubated at 37 °C and 5% CO_2_ for 1 h. After washing thrice with 1×PBS, observation and image collection were performed using fluorescent microscopy.

### 2.7 Cell culture

The mouse RAW264.7 macrophages were cultured in DMEM (Gibco, United States) with 10% FBS, 100 U/mL penicillin, and 100 mg/mL streptomycin and maintained under standard culture conditions.

### 2.8 RAW264.7 macrophages uptake hucMSC-EV

We used live-cell imaging to verify hucMSC-EV uptake by RAW264.7 macrophages. First, a working solution was prepared by mixing 100 μg hucMSC-EV, 0.1 mL Alexa Fluor 488 dye, and 10 mmol sodium carbonate in 1 mL 1×PBS, followed by incubation with RAW264.7 cells. Images were obtained at 3 h using confocal microscopy (FV300; Olympus, Japan).

CellTracker™ Red CMTPX Dye (C34552, ThermoFisher, United States) can enter and stay in live cells, and can emit 602 nm light excitated by 577 nm excitating light. Incubated EV with CellTracker™ Red CMTPX Dye for 30 min at 37°C, and centrifuged the EV to remove the uncombined CellTracker™ working solution. Added the labeled EV into RAW264.7 cells and took images with inverted fluorescence microscope (DMi8, Leica, Germany). If RAW264.7 cells could uptake exosoems, we can see red light in RAW264.7 cells.

### 2.9 Quantitative PCR

RAW264.7 cells (1 × 10^6^) were seeded in 6-well dishes. When 40% confluency was reached, the medium was replaced with complete DMEM (containing 10% FBS, 100 U/mL penicillin, and 100 mg/mL streptomycin), and, for the LPS group, 100 ng/mL LPS. After 8 h, the medium was replaced with fresh complete DMEM, in which 20 µL DMEM or DPBS or 1 mg/mL hucMSC-EV solution was added. Cells were collected after 12 h. Total RNA was extracted using a RaPure Total RNA Micro Kit (R4012-02; Magen, Shanghai) according to standard protocols. cDNA (from 1 μg mRNA) was generated using the Fast All-in-One RT Kit (RT001; ES Science, Shanghai). mRNA expression was assessed using quantitative (qRT)–PCR with SYBR qPCR Mix (Q311; Vazyme, Nanjing) in Bio-Rad CFX996 and analyzed using the Bio-Rad manager software (Bio-Rad, Hercules, CA, United States) normalized to GAPDH. Results were calculated using the 2^−ΔΔCT^ method. cDNA was then amplified *via* PCR using the primer sequences listed in Supplementary Table S1.

### 2.10 ELISA

We used an ELISA kit to measure the levels of TNF-α, IL-1β, and IL-10 (KE10002, KE10003, KE10008; Proteintech, United States of America) in the culture supernatant. The experimental method follows that described for qRT-PCR. After adding hucMSC-EV for 48 h, the culture supernatant was collected. Following centrifugation at 500 × *g* for 5 min, the culture supernatant was stored at −20°C. The microplate strips were removed, and the microwells were placed in the strip holder. One hundred microliters of each sample were added to the appropriate wells, and the plate was incubated for 2 h at 37°C in a humid environment. After washing the wells 4 times with 1 × Wash Buffer, 100 μL of 1 × antibody detection solution was added, followed by incubation for 1 h at 37°C in a humid environment. After washing, 100 μL of 1 × HRP-conjugated antibody was added to each well, followed by incubation for 40 min at 37°C in a humid environment. After washing, 100 μL of TMB substrate solution was added to each well, followed by incubation for 15 min at 37°C in the dark. Stop solution (100 μL) was added, and absorbance at 450 nm was read immediately on a microplate reader. (BioTek, Synergy H1M) Cytokine concentrations were calculated according to the standard curve.

### 2.11 Statistical analysis

Data were processed using SPSS 20.0 statistical software (IBM, Armonk, NY, United States). The data are shown as the mean ± standard deviation from at least three independent experiments. Comparisons among multiple groups were performed using one-way analysis of variance (ANOVA) and Tukey’s test. Data between two groups were compared using the unpaired t-test or Kruskal–Wallis test. Statistical significance was set at *p < .05*.

## 3 Results

### 3.1 Extraction and identification of HucMSCs

**FIGURE 1:**
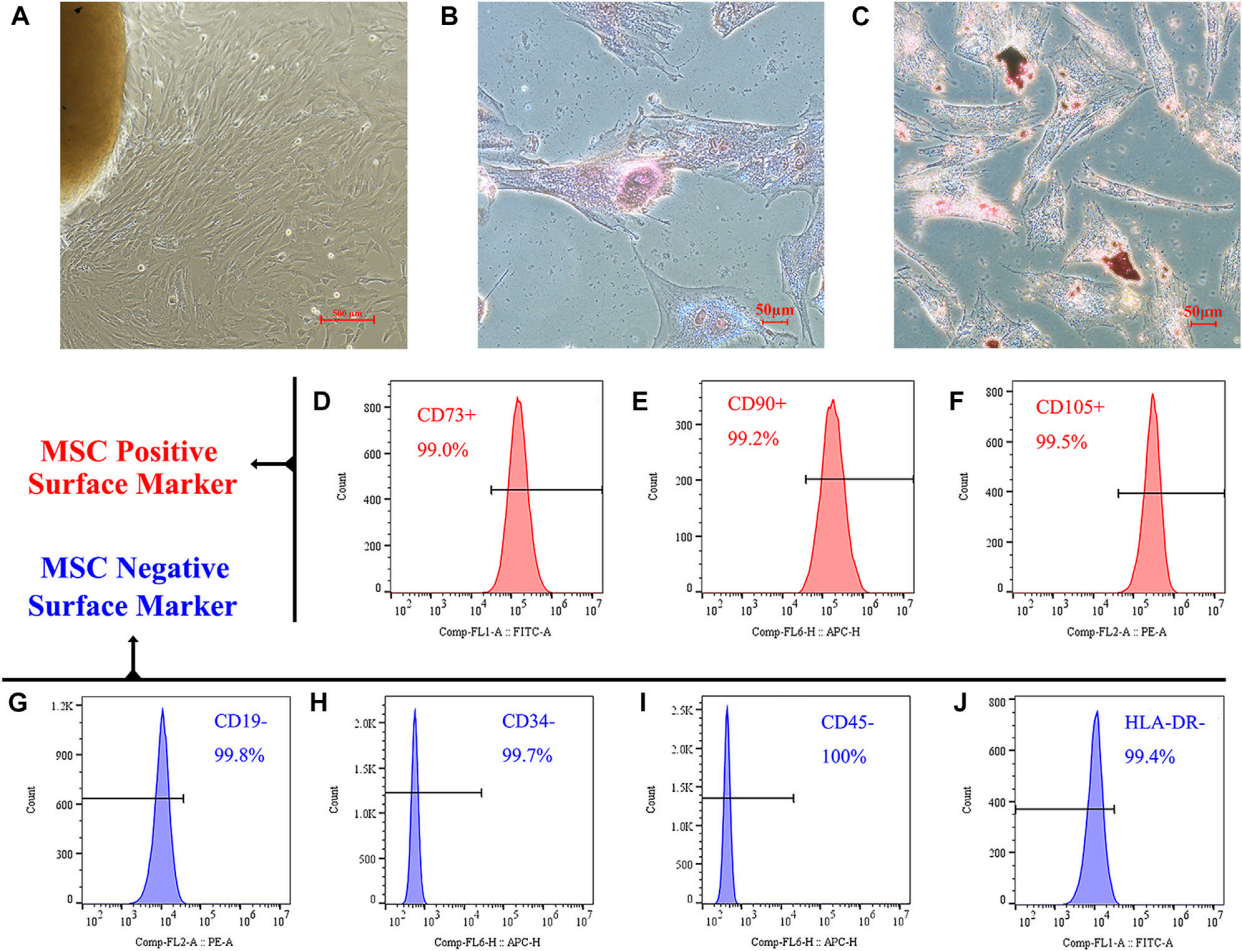
Identification of hucMSCs. **(A)** HucMSCs were isolated and purified through their physical adherence to the plastic cell culture plate. **(B)** Alizarin red S staining demonstrated that mineralized nodules were formed in the hucMSCs 3 weeks after osteogenic induction. **(C)** Oil-red-O staining showed lipid-rich vacuole formation in the mouse BM-MSCs after 3 weeks of adipogenic induction. **(D–F)** Flow cytometry analysis showed that these cells were positive for MSC markers CD73 **(D)**, CD90 **(E)**, and CD105 **(F)**. **(G–J)** Cells were negative for B cell marker CD19 **(G)**, endothelial cell marker CD34 **(H)**, pan-leukocyte marker CD45 **(I)**, and stimulated immune cell marker HLA-DR **(J)**.

The high quality of the extracted hucMSCs was identified based on three properties: adherence capacity, differentiation ability, and presence of surface markers, according to the criteria proposed by the mesenchymal and tissue stem cell Committee of the International Society for Cellular Therapy. As shown in Figure 1A, MSCs climbed outward from the central umbilical cord block and could adhere to the wall for growth. Osteogenic and adipogenic experiments verified the differentiation potential of the cells. After a 3-week induction and culture in an osteogenic differentiation medium, MSCs successfully differentiated into osteoblasts (Figure 1B); calcium deposition could be observed using ARS staining. After a 3-week induction and culture with an adipogenic differentiation medium, MSCs successfully differentiated into adipocytes (Figure 1C). Using oil red O staining, red lipid droplets in the cells were observed.

We further verified that our isolated and cultured cells were MSCs using flow cytometry. As shown in Figures 1D–F, the positive markers, CD73, CD90, and CD105, on the surface of MSCs were expressed in >99% of the cells. Figures 1G–J shows that the expression rate of MSC negative markers and exclusion proteins, CD19, CD34, CD45, and HLA-DR, on the cell surface was <1%.

### 3.2 Identification of hucMSC-derived EV

**FIGURE 2:**
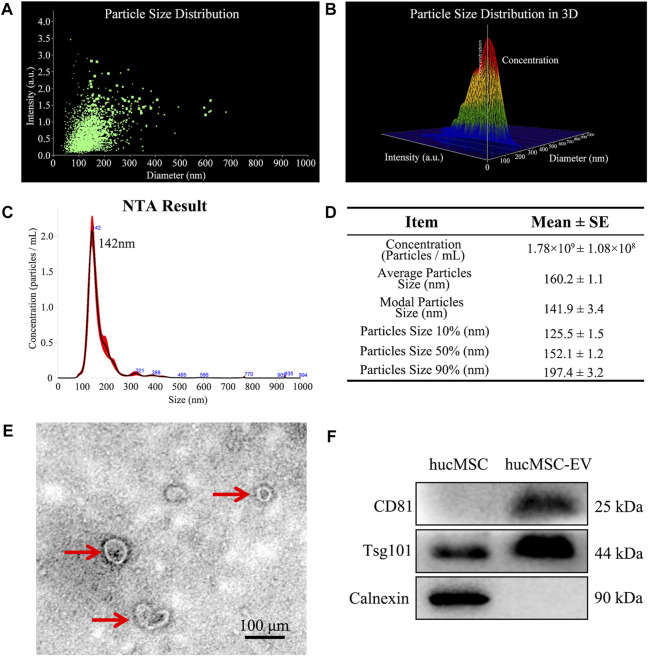
Identification of hucMSC-EV. **(A, B)** Size distribution and schematic illustration of hucMSC-EV measured from NanoSight. **(C)** NanoSight measure of the particle diameter of hucMSC-EV. **(D)** A table for particle concentration, average particle size and modal particle size. **(E)** Transmission electron micrographs of hucMSC-EV. **(F)** Western blotting identified the positive exosome protein markers CD81 and Tsg101 and negative exosome protein marker Calnexin.

To evaluate whether the extracted EV conformed to international standards, we separated and extracted extracellular vesicles using ultracentrifugation. Figures 2A–D showed size distribution and schematic illustration of hucMSC-EV measured from NanoSight and then we observed isolated structures using projection electron microscopy. The results are shown in Figure 2E. We observed a double concave disc-shaped vesicle structure with a diameter of ∼100 nm. Using NTA, the peak particle size of extracellular vesicles was estimated at 142 nm; particle size was within the 40–100 nm range and complied with international standards (Figure 2C). Further verification using western blotting (WB) showed that the extracellular vesicle structure expressed the membrane protein CD81 and intracellular protein TSG101 but not calnexin (located in the endoplasmic reticulum). In contrast, hucMSCs expressed TSG101 and calnexin, and the expression of CD81 was substantially lower than that of the extracted MSC extracellular vesicle structure (Figure 2F). Therefore, we successfully extracted hucMSC-EV that conformed to international standards.

### 3.3 hucMSC-EV alleviated tubal factor infertility caused by CT infection and increased the pregnancy rate of nude mice

**FIGURE 3:**
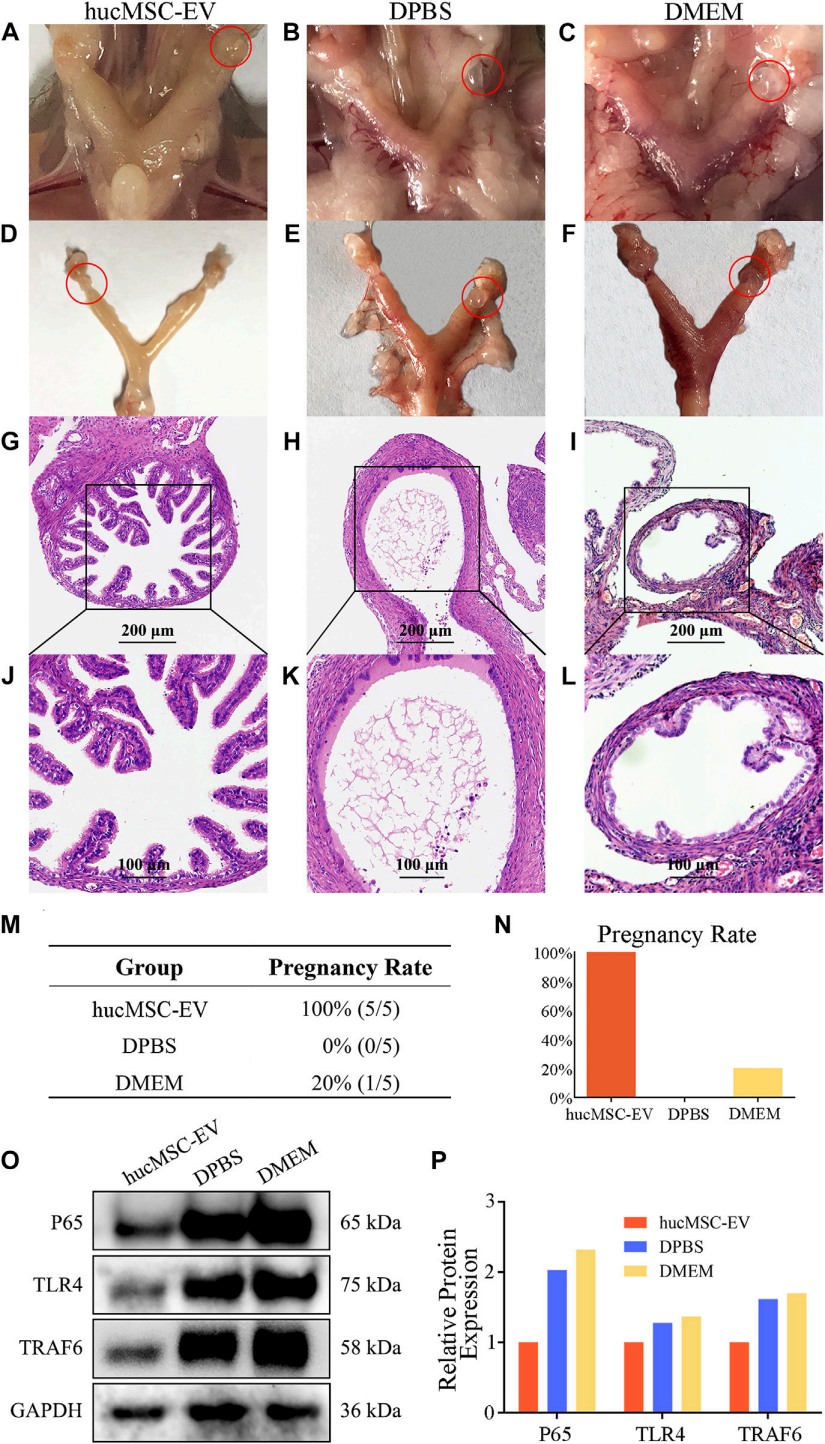
The therapeutic effect of hucMSCs on a murine model of chronic salpingitis and molecular mechanism. **(A–L)** Representative photographs and H&E microphotographs of mice fallopian tubes in the murine chronic salpingitis model. **(M, N)** The pregnancy rate table and graph of each group. **(O, P)** Representative western blots of P65 and TLR4 in the mice fallopian tubes from each group **(O)** and the relative protein expression **(P)**; band intensities were normalized against the corresponding GAPDH.

We successfully induced a mouse model of tubal inflammatory infertility caused by CT infection. To verify that hucMSC-EV can alleviate salpingitis and improve the pregnancy rate in pathogenic animals, mice were first divided into hucMSC-EV, DPBS, and DMEM groups, as shown in Figures 3A–F. After hucMSC-EV treatment, local hyperemia and the inflammatory hydrosalpinx in the mouse oviduct and uterus were considerably reduced compared with those in the control group. In contrast, local hyperemia and hydrosalpinx were observed in the DPBS and DMEM groups. H&E tissue staining showed that the structure of oviduct villi in the hucMSC-EV group was complete, while the villi structure in the DPBS and DMEM groups almost disappeared, with evident tissue expansion due to ponding (Figure 3G−L). The above results showed that hucMSC-EV can significantly improve reproductive tract congestion and the inflammatory response in pathogenic mice.

In the next experiment, we examined whether the pregnancy rate in these mice could be improved. As shown in Figures 3M, N, all five mice in the hucMSC-EV group were pregnant, compared with none in the DPBS group and one in the DMEM group. The above results further verified that the application of hucMSC-EV could significantly improve the reproductive tract congestion and inflammatory response of pathogenic mice, thereby improving the pregnancy rate.

To further explore the molecular mechanism and signaling pathway of hucMSC-EV in alleviating the inflammatory response in pathogenic mice, we detected the proteins p65 and TLR4 related to the inflammatory signaling pathway. We prepared protein samples and carried out WB analysis using oviduct tissues from the three mice groups (Figures 3O, P). The expression levels of p65 and TLR4 in the hucMSC-EV group were significantly lower than those in the DPBS and DMEM groups. These results suggest that p65 and TLR4 may play an important role in the hucMSC-EV-mediated alleviation of oviduct inflammation.

### 3.4 hucMSC-EV induced macrophage polarization from M1 to M2

**FIGURE 4:**
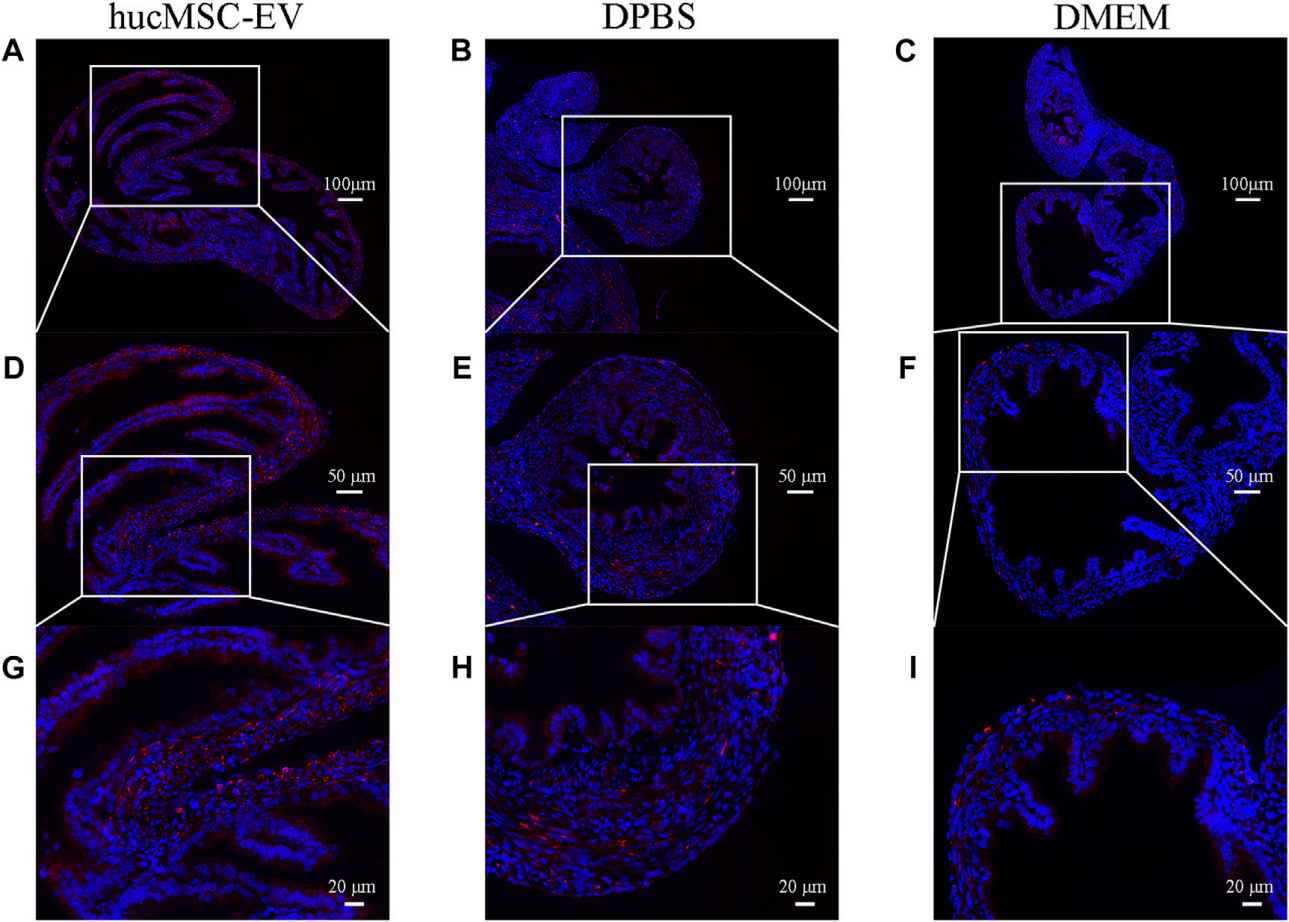
Immunofluorescence of CD206 showing M2 macrophages in the murine fallopian tubes in each experimental group **(A-I)**. Immunofluorescence of CD206 (red) showing M2 macrophages in the murine fallopian tubes in hucMSC-EV group **(A, D, G)**, DPBS group **(B, E, H)**, and DMEM group **(C, F, I)**.

To explore whether hucMSC-EV play a crucial role in tubal factor infertility by regulating macrophage polarization, we labeled CD206 with immunofluorescence to show the distribution of M2 macrophages in tubal tissue. The results revealed (Figure 4) that the number of M2 macrophages in the hucMSC-EV group was considerably higher than that in the DPBS and DMEM groups, suggesting that hucMSC-EV may inhibit the oviduct inflammatory response caused by *chlamydia* infection by inducing macrophage polarization from the M1 to M2 type.

### 3.5 Macrophages ingest membrane components of HucMSC culture supernatant and hucMSC-EV *in vitro*

**FIGURE 5:**
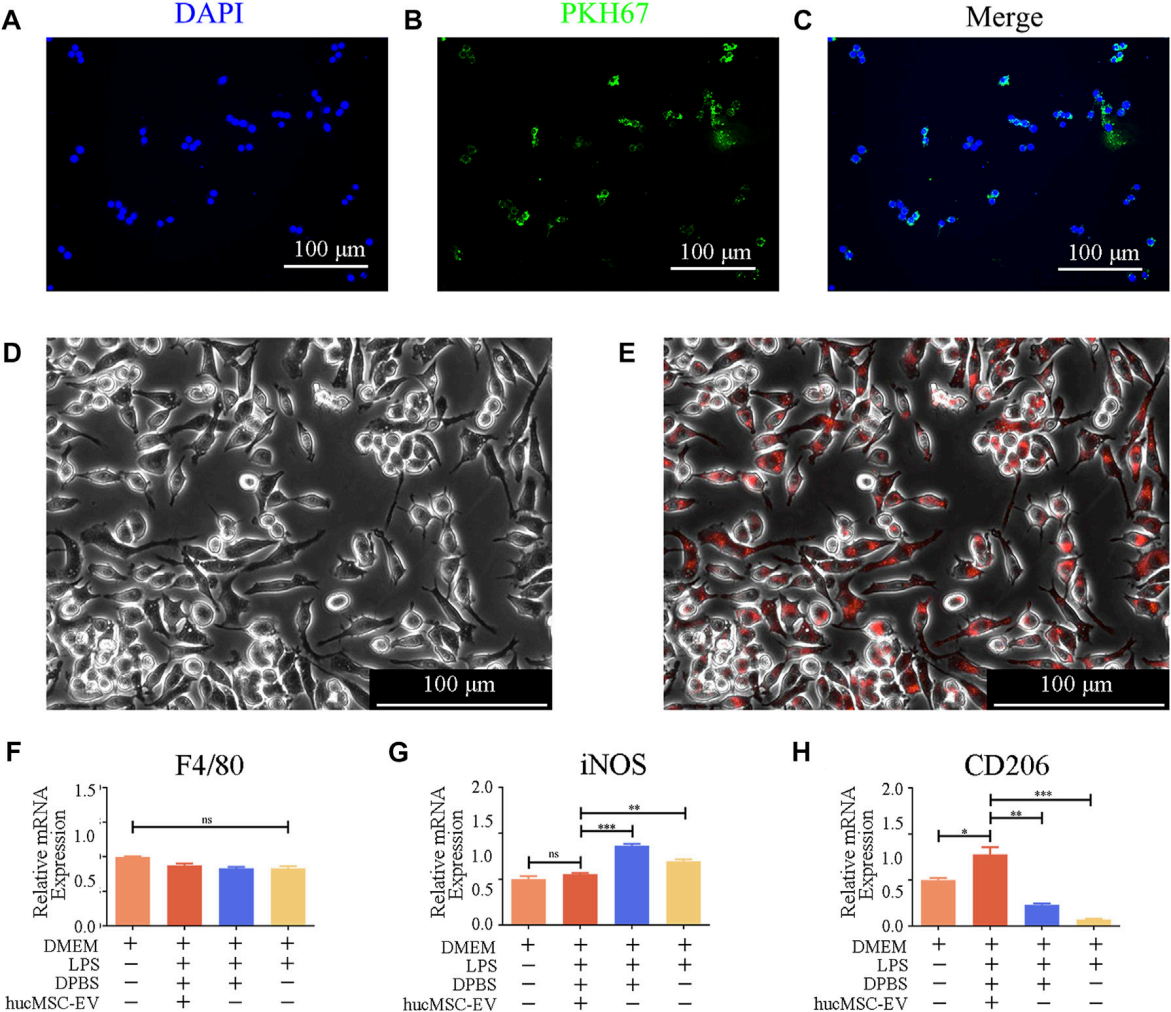
hucMSC-EV uptake by mouse RAW264.7 macrophages and hucMSC-EV-mediated polarization of RAW264.7 cells. **(A–C)** Immunofluorescence image showing the uptake of membrane components of hucMSC culture supernatant by RAW264.7 cells. **(D, E)** Immunofluorescence image indicating RAW264.7 cell uptake of hucMSC-EV. **(F–H)** Gene expression profiles of macrophage subtype markers in RAW264.7 cells in each group (*n* = 3). Data are expressed as the mean ± SD. Statistical significance was determined using one-way ANOVA followed by an unpaired t-test or Kruskal–Wallis test. *p < .05, **p < .01, ***p < .001.

How do hucMSC-EV induce macrophages to polarize from M1 cells to M2 cells to alleviate salpingitis? To further explore the molecular mechanism of the macrophage program, we conducted the following experiments. As shown in Figures 5A–C, we first labeled membrane components in the hucMSC culture supernatant using PKH67 (green fluorescence) and nuclei using DAPI (blue fluorescence). After fusion, we found that the green fluorescence signal (model component) surrounded the blue nuclei, indicating that macrophage RAW264.7 cells can swallow membrane components in the hucMSC culture supernatant.

Additionally, we used CellTracker™ Red CMTPX Dye to label EV. Fluorescent microscopy showed that the red signal coincided with macrophages, verifying that macrophages could phagocytose EV (Figures 5D,E).

Next, we explored whether hucMSC-EV could reverse the M1-type macrophages to the M2 type.

We first treated macrophages separately with LPS and hucMSC-EV and detected the surface marker proteins of M1 and M2 macrophages using RT-PCR to verify the role of hucMSC-EV. As shown in Figures 5F–H, after treatment with 100 ng/ml of LPS, the transcription level of iNOS in M1 macrophages increased significantly, while that of CD206 in M2 macrophages decreased significantly. When hucMSC-EV and LPS were present simultaneously, the transcription level of iNOS in M1 macrophages was not significantly different from that in the untreated group, while the transcription level of CD206 in M2 macrophages was significantly different from that in the other three groups. LPS and hucMSC-EV had no significant effect on the expression of macrophage general marker F4/80. The above results indicate that hucMSC-EV can reverse LPS-induced macrophage transformation and promote the conversion of M1-type to M2-type macrophages.

### 3.6 hucMSC-EV reverse p65 and TRAF6 expression in LPS-induced macrophages and promote IL-10 transcription

**FIGURE 6:**
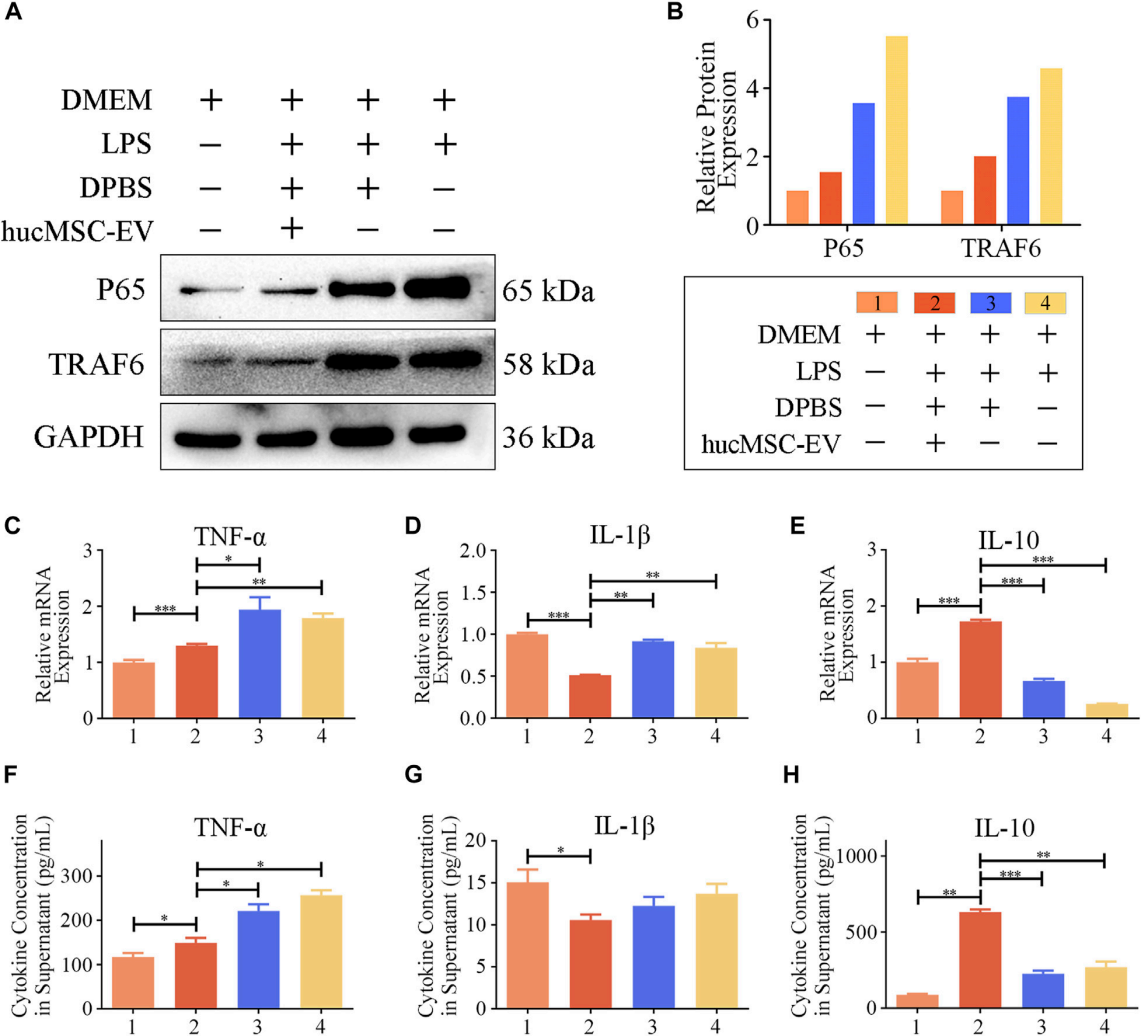
hucMSC-EV regulate p65, TRAF6, and cytokine transcription and expression. **(A, B)** Representative western blots of p65 and TRAF6 in RAW264.7 cells from each group **(A)** and the relative protein expression **(B)**; band intensities were normalized against the corresponding GAPDH. **(C–E)** mRNA expression levels of cytokine genes in RAW264.7 cells treated with LPS for 8 h and hucMSC-EV for 12 h (*n* = 3). **(F–H)** Concentration of cytokines TNF-α **(F)**, IL-1β **(G)**, and IL-10 **(H)** in the culture supernatant of RAW264.7 cells treated with LPS for 8 h and hucMSC-EV for 48 h (*n* = 4). Data are expressed as the mean ± SD. Statistical significance was determined using one-way ANOVA followed by an unpaired t-test or Kruskal–Wallis test. *p < .05, **p < .01, ***p < .001.

To further explore the molecular mechanism by which hucMSC-EV induce macrophage polarization, we treated macrophages with hucMSC-EV and LPS in vitro cell experiments and verified the expression of p65 and TRAF6 proteins using WB. As shown in Figures 6A,B, LPS significantly upregulated the expression levels of p65 and TRAF6, while simultaneous treatment of macrophages with LPS and hucMSC-EV did not result in significant increases in the expression levels of p65 and TRAF6. These results indicate that hucMSC-EV can reverse these processes through the p65/TRAF6 pathway.

M1 macrophages usually secrete proinflammatory cytokines and IL-1β, while M2 macrophages usually secrete anti-inflammatory cytokine IL-10. Could hucMSCs promote the expression of anti-inflammatory cytokine IL-10? The results show (Figures 6C–H) that LPS treatment significantly increased macrophage TNF-α and IL-1β mRNA transcription and secretion levels, whereas treatment with hucMSC-EV downregulated the transcription and expression levels of both factors. Furthermore, LPS significantly reduced the mRNA transcription and secretion level of IL-10 in macrophages, while hucMSC-EV significantly upregulated the transcription and expression level of IL-10. The above results show that hucMSC-EV can reverse the expression of p65 and TRAF6 proteins in macrophages induced by LPS and promote the transcription and secretion of IL-10.

## 4 Discussion

**FIGURE 7:**
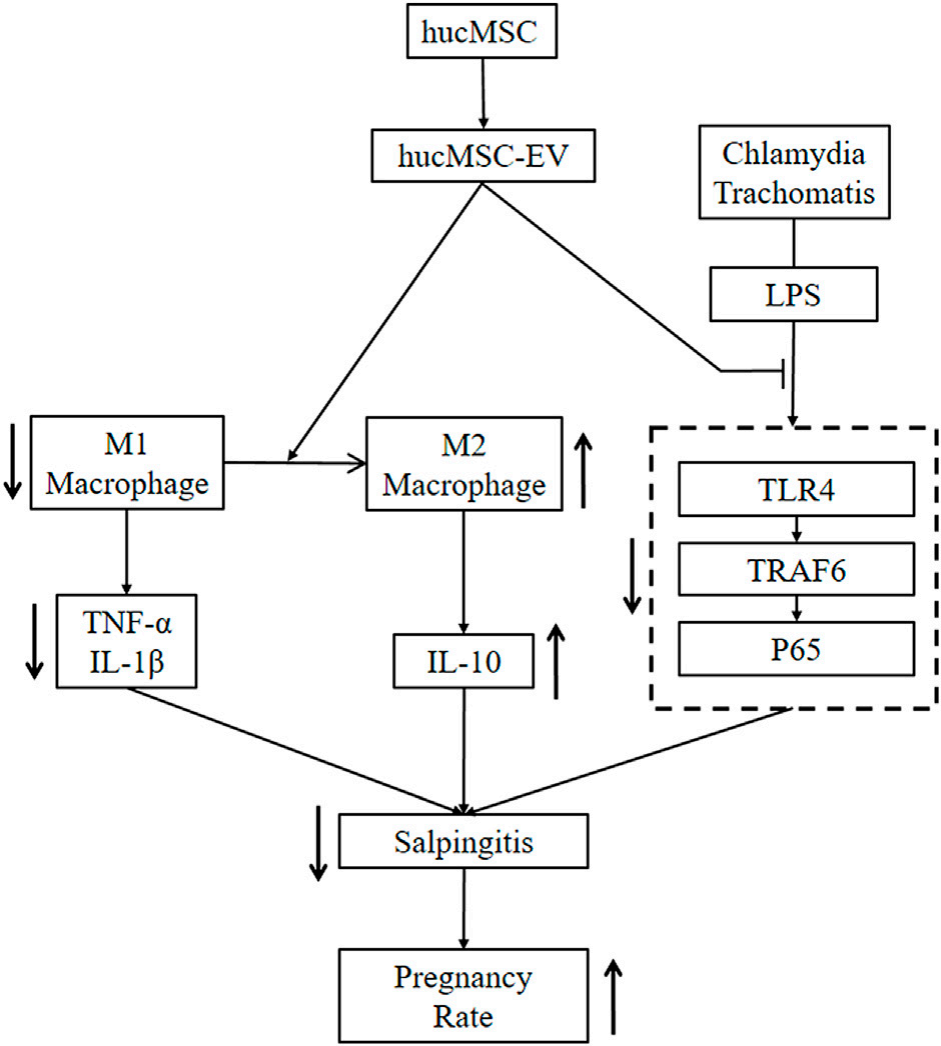
hucMSC-EV treat salpingitis by promoting macrophage polarization from M1 to M2 and inhibiting the TLR4 signaling pathway. hucMSC-EV downregulated M1 macrophages and cytokines TNF-α and IL-1β and upregulated M2 macrophages and cytokine IL-10. hucMSC-EV downregulated the TLR4, TRAF6, and p65 proteins to treat salpingitis and upregulated the pregnancy rate.

WHO has predicted that infertility will become the third major disease in the 21st century (Mascarenhas et al., 2012). The most common cause of infertility is salpingitis caused by CT, with up to 70% of patients being asymptomatic (Yonke et al., 2022); lack of appropriate treatment in undiagnosed cases can lead to infertility (Hafner, 2015). Here, we demonstrated the functional significance of hucMSC-derived EV in infertility caused by CT-induced salpingitis *in vitro* and *in vivo* and the possible mechanisms of hucMSC-EV. hucMSC-EV reduced the congestion and inflammation in fallopian tubes caused by the CT infection and increased the pregnancy rate of nude mice. Regarding the molecular mechanism, we found that hucMSC-EV, through the NF-κB signaling pathway, can induce macrophages to transform from the M1 to the M2 type and inhibit tubal factor infertility (Figure 7). These results lay a solid foundation for the future clinical applications of EV to inhibit CT inflammatory infertility.

EV can be obtained from a variety of specimens, including cell culture supernatants and various body fluids, such as blood and urine (Logozzi et al., 2020). MSC-derived EV play an increasingly important role in intracellular communication and tissue repair (Joo et al., 2020; Lin et al., 2022). Compared with living cells, their clinical application may offer significant advantages because they may reduce adverse side effects after application, as well as infusion toxicity, uncontrolled cell growth, and possible tumor formation (Liang et al., 2014; Qiu et al., 2019; Weng et al., 2021).

The use of hucMSC-EV has shown beneficial effects in a variety of diseases, such as lung disease (Willis et al., 2020; Willis et al., 2021), oxygen-Induced multi-organ disease (Fernandez-Gonzalez et al., 2021), preeclamptic lung Injury (Taglauer et al., 2022), various detrimental sequalae of hyperoxia exposure (Reis et al., 2021), cornea disease (McKay et al., 2021), hepatic fibrosis (Rong et al., 2019), *etc.* In rat calvarial defects, miR-196a (Qin et al., 2016), miR-27a, and miR-206 (Lai et al., 2022) positively regulate the expression of osteogenic genes and osteoblast differentiation to stimulate bone formation (Liu et al., 2018). In a mouse model of Alzheimer’s disease, miR-21 and miR-181c effectively reduced amyloid-β accumulation and increased synaptic protein expression and miR-21 levels in the brains of APP/PS1 mice (Cui et al., 2018). EV inhibited the hypoxic activation of STAT3 signaling and upregulation of miR-204 and miR-17 in a mouse model of suppressed pulmonary hypertension (Lee et al., 2012). Inhibition of the IL-6-related signaling pathway by miR-455-3p in a mouse model of acute liver injury suppressed monocyte/macrophage overactivation and reduced injury (Shao M et al., 2020). In a rat model of acute kidney injury, the renal injury was suppressed by improving oxidative stress and apoptosis, and cell proliferation was promoted by activation of ERK1/2 *in vivo* and *in vitro* (Ullah et al., 2020). Inhibition of myofibroblast formation and TGF by miR-21, miR-23a, miR-125b, and miR-145 in a mouse model of skin disease- β 2, TGF- β R2 and Smad2 pathways, thereby inhibiting α-SMA expression and decreasing collagen I deposition (Hu et al., 2020). EV act on TRAF1-mediated macrophage polarization, thereby treating severe steroid-resistant asthma. Treatment of cerebral ischemic injury with miR-542-3p prevented ischemia-induced glial inflammatory response by inhibiting TLR4 (Cai et al., 2021). Many studies have also explored the use of hucMSC-EV or their secretomes to treat COVID-19 infection with promising results (Meng et al., 2020; Sengupta et al., 2020; Tang et al., 2020; Hashemian et al., 2021).

At present, MSCs are rarely used in the treatment of tubal factor infertility. Our previous study showed that MSC transplantation can significantly reduce the degree of inflammation in salpingitis caused by CT infection (Liao et al., 2019). In this study, we demonstrate that hucMSC-EV can reduce salpinx congestion and inflammatory reactions caused by CT infection and improve the pregnancy rate of nude mice. Therefore, we speculate that the role of MSCs in the repair and reconstruction of inflammatory adhesions may be related to the immunoregulatory effects of MSCs under different inflammatory environments and on other inflammatory cells.

Some studies have confirmed that MSCs can promote the transformation of M1 macrophages to the M2 type through the paracrine pathway (Xin et al., 2020; Li et al., 2022; Teng et al., 2022; Zhang et al., 2022), suggesting that M2-type macrophages are important in the MSC-mediated treatment of salpingitis. Based on previous studies, we used CT intravaginal inoculation to generate a mouse model of salpingitis. hucMSC-EV were used to treat CT salpingitis, and the therapeutic effects were evaluated. We found that membrane components in the supernatant of hucMSCs could be engulfed by M1 macrophages, inducing M2-type polarization. Furthermore, the number of M2 macrophages in the hucMSC-EV group was substantially higher than that in the other groups, suggesting that hucMSC-EV can inhibit tubal inflammation caused by chlamydial infection by inducing macrophage polarization from the M1 to M2 type. However, the molecular mechanism by which hucMSC-EV induce macrophage polarization remains unknown.

NF-κB is a major transcription factor with a key role in the immune response (Xu et al., 2019; Barnabei et al., 2021; Luo et al., 2022). Strict regulation of the NF-κB signaling pathway is essential for maintaining immune homeostasis (Mitchell and Carmody, 2018; Yu et al., 2020). Uncontrolled hyperactivation of this pathway may lead to excessive inflammation and ultimately to various pathological conditions (Taniguchi and Karin, 2018).

NF-κB acts *via* two signaling pathways: a typical pathway that mediates inflammatory responses (Barnabei et al., 2021) and an atypical pathway that participates in the differentiation and maturation of immune cells (Hayden and Ghosh, 2011) and secondary lymphoid organogenesis (Hahn et al., 2016; Sun, 2017). Inhibition of NF-κB activation can promote immunosuppression in inflammation and tumors (Li et al., 2020); in contrast, stimulation of NF-κB activation promotes immunity and activates CD8^+^ T cell cytotoxicity (Yatim et al., 2015). In patients with multiple sclerosis, related gene alterations led to the enhancement of the NF-κB signaling pathway, enhancing the inflammatory response (Leibowitz and Yan, 2016). The degradation of NF that induces autophagy κ B-rela cells, restoring NF κ After B activity, tumor-associated macrophages can be seen to polarize toward M2 (Qian M et al., 2020; Korbecki et al., 2021; Zhang et al., 2022).

Inhibition of the nuclear translocation of NF-κB leads to decreased transcriptional activity (Yu et al., 2020); when its phosphorylation is inhibited, the inflammatory response is suppressed (Baldwin, 2012). Valsartan is the latest generation of angiotensin II receptor antagonists, which can reduce NF-κB nuclear translocation, decrease its expression, inhibit NF-κB-related inflammatory pathways, and reduce the expression of the inflammatory end products COX-2 and IL-1 (Dandona et al., 2003; Sun et al., 2018). In addition, inhibition of the NF-κB p65 signaling pathway can inhibit TNF-α-induced expression of inflammatory mediators (Deng et al., 2010). The LPS-induced NF-κB signaling pathway can be selectively regulated to inhibit the expression of proinflammatory genes, thereby inhibiting the inflammatory response (Gao et al., 2022). In addition, Mammalian sterile 20-like kinase 1 (MST1) attenuates NF-κB-dependent inflammatory gene expression by phosphorylating HOIP and, thus, functions as a negative regulatory mechanism, promoting the regression of inflammation and preventing unnecessary tissue damage (In Young Lee et al., 2019).

To further explore the molecular mechanism and signaling pathways of hucMSC-EV in reducing inflammation in pathogenic mice, we detected the proteins p65 and TLR4, associated with the inflammatory signaling pathway. The results show that the protein expression levels of p65 and TLR4 were significantly downregulated in hucMSC-EV, suggesting that p65 and TLR4 may play an important role in the hucMSC-EV-mediated treatment of salpingitis. Subsequently, we found that hucMSC-EV can reverse the expression of p65 and TRAF6 proteins in LPS-induced macrophages and promote the transcription and secretion of IL-10; therefore, hucMSC-EV can regulate the NF-κB signaling pathway and induce macrophage-polarization from M1 to M2, improving the local inflammatory microenvironment of fallopian tubes.

Although we found that hucMSC-EV can downregulate the NF-κB inflammatory signaling pathway, contributing to the reduction of inflammation, several factors remain unknown: 1) identification of the upstream molecules in the TLR4 signaling pathway; 2) secretions comprise several substances, including RNA, proteins, and other biological molecules, with yet unresolved roles. In this regard, future investigations using chip technology and bioinformatics may be useful; 3) the potential anti-inflammatory effects and synergistic actions of substances in the culture supernatant of hucMSCs, such as growth factors and other types of cell vesicles, require investigation; 4) in addition, application of hucMSC-EV to studies of infertility caused by other factors, such as endometritis caused by CT (Yang et al., 2021), remains open. The above issues remain the direction and goal of our future studies, and our efforts will be toward designing appropriate experiments.

## 5 Conclusion

CT infection can cause chronic salpingitis and infertility. hucMSC-EV can induce macrophage polarization from the M1 to M2 type through the NF-κB signaling pathway, thus improving the local inflammatory microenvironment of the fallopian tube, treating chronic salpingitis caused by CT infection, and ultimately improving the reproductive outcome. Thus, hucMSC-EV can be a promising, cell-free method to treat infertility due to chronic salpingitis.

## Data Availability

The original contributions presented in the study are included in the article/Supplementary Material, further inquiries can be directed to the corresponding authors.

## Abbreviations

CT, *Chlamydia trachomatis*; MSCs, Mesenchymal stem cells; MSC-EV, Mesenchymal stem cells-derived extracellular vesicles; MSC-CM, Mesenchymal stem cells-conditional medium; hucMSC-EV, human umbilical cord mesenchymal stem cell-derived extracellular vesicles; WHO, World Health Organization; MST1, Mammalian sterile 20-like kinase 1

## References

[B1] Andrzejewska A. Lukomska B. Janowski M. (2019). Concise Review: Mesenchymal stem cells: From roots to boost. Stem Cells Dayt. Ohio) 37 (7), 855–864. 10.1002/stem.3016 PMC6658105 30977255

[B2] Baldwin A. S. (2012). Regulation of cell death and autophagy by IKK and NF-?b: Critical mechanisms in immune function and cancer. Immunol. Rev. 246 (1), 327–345. 10.1111/j.1600-065X.2012.01095.x 22435564

[B3] Barnabei L. Laplantine E. Mbongo W. Rieux-Laucat F. Weil R. (2021). NF-ΚB: At the borders of autoimmunity and inflammation. Front. Immunol. 12, 716469. 10.3389/fimmu.2021.716469 34434197 PMC8381650

[B4] Brunham R. C. (2022). Problems with understanding Chlamydia trachomatis immunology. J. Infect. Dis. 225 (11), 2043–2049. 10.1093/infdis/jiab610 34919679

[B5] Cai G. Cai G. Zhou H. Zhuang Z. Liu K. Pei S. (2021). Mesenchymal stem cell-derived exosome MiR-542-3p suppresses inflammation and prevents cerebral infarction. Stem Cell. Res. Ther. 12 (1), 2. 10.1186/s13287-020-02030-w 33407827 PMC7786953

[B6] Cooke J. P. (2019). Inflammation and its role in regeneration and repair. Circulation Res. 124 (8), 1166–1168. 10.1161/CIRCRESAHA.118.314669 30973815 PMC6578588

[B7] Cui G.-H. Wu J. Mou F.-F. Xie W.-H. Wang F.-B. Wang Q.-L. (2018). Exosomes derived from hypoxia-preconditioned mesenchymal stromal cells ameliorate cognitive decline by rescuing synaptic dysfunction and regulating inflammatory responses in APP/PS1 mice. FASEB J. Official Publ. Fed. Am. Soc. Exp. Biol. 32 (2), 654–668. 10.1096/fj.201700600R 28970251

[B8] Dandona P. Kumar V. Aljada A. Ghanim H. Syed T. Hofmayer D. (2003). Angiotensin II receptor blocker valsartan suppresses reactive oxygen species generation in leukocytes, nuclear factor-kappa B, in mononuclear cells of normal subjects: Evidence of an antiinflammatory action. J. Clin. Endocrinol. Metabolism 88 (9). 10.1210/jc.2002-021836 12970329

[B9] Deng N. Ye Y. Wang W. Li L. (2010). Dishevelled interacts with P65 and acts as a repressor of NF-ΚB-Mediated transcription. Cell. Res. 20 (10), 1117–1127. 10.1038/cr.2010.108 20628365

[B10] Dominici M. Le Blanc K. Mueller I. Slaper-Cortenbach I. Marini F. Krause D. (2006). Minimal criteria for defining multipotent mesenchymal stromal cells. The international society for cellular Therapy position statement. Cytotherapy 8 (4), 315–317. 10.1080/14653240600855905 16923606

[B11] Erpicum P. Rowart P. Poma L. Krzesinski J.-M. Detry O. Jouret F. (2017). Administration of mesenchymal stromal cells before renal ischemia/reperfusion attenuates kidney injury and may modulate renal lipid metabolism in rats. Sci. Rep. 7 (1), 8687. 10.1038/s41598-017-08726-z 28819187 PMC5561049

[B12] Farhat W. Yeung V. Kahale F. Parekh M. Cortinas J. Chen L. (2022). Doxorubicin-loaded extracellular vesicles enhance tumor cell death in retinoblastoma. Bioeng. (Basel, Switz. 9 (11), 671. 10.3390/bioengineering9110671 PMC9687263 36354582

[B13] Fernandez-Gonzalez A. Willis G. R. Yeung V. Reis M. Liu X. Mitsialis S. A. (2021). Therapeutic effects of mesenchymal stromal cell-derived small extracellular vesicles in oxygen-induced multi-organ disease: A developmental perspective. Front. Cell. Dev. Biol. 9, 647025. 10.3389/fcell.2021.647025 33796534 PMC8007882

[B14] Galipeau J. (2021). Macrophages at the nexus of mesenchymal stromal cell potency: The emerging role of chemokine cooperativity. Stem Cells Dayt. Ohio) 39 (9), 1145–1154. 10.1002/stem.3380 PMC8453730 33786935

[B15] Gao Y. Xu Z. Li X. Liu Z. Li W. Kang Y. (2022). Resibufogenin, one of bufadienolides in toad venom, suppresses LPS-induced inflammation via inhibiting NF-?b and AP-1 pathways. Int. Immunopharmacol. 113, 109312. 10.1016/j.intimp.2022.109312 36252491

[B16] Hafner L. M. (2015). Pathogenesis of fallopian tube damage caused by Chlamydia trachomatis infections. Contraception 92 (2), 108–115. 10.1016/j.contraception.2015.01.004 25592078

[B17] Haggerty C. L. Gottlieb S. L. Taylor B. D. Low N. Xu F. Ness R. B. (2010). Risk of sequelae after Chlamydia trachomatis genital infection in women. J. Infect. Dis. 201 (2), S134–S155. 10.1086/652395 20470050

[B18] Hahn M. Macht A. Waisman A. Hövelmeyer N. (2016). NF-ΚB-Inducing kinase is essential for B-cell maintenance in mice. Eur. J. Immunol. 46 (3), 732–741. 10.1002/eji.201546081 26593098

[B19] Harrell C. R. Jovicic N. Djonov V. Arsenijevic N. Volarevic V. (2019). Mesenchymal stem cell-derived exosomes and other extracellular vesicles as new remedies in the Therapy of inflammatory diseases. Cells 8 (12), 1605. 10.3390/cells8121605 31835680 PMC6952783

[B20] Hashemian S.-M. R. Aliannejad R. Zarrabi M. Soleimani M. Vosough M. Hosseini S.-E. (2021). Mesenchymal stem cells derived from perinatal tissues for treatment of critically ill COVID-19-induced ards patients: A case series. Stem Cell. Res. Ther. 12, 91. 10.1186/s13287-021-02165-4 33514427 PMC7844804

[B21] Hayden M. S. Ghosh S. (2011). NF-ΚB in immunobiology. Cell. Res. 21 (2), 223–244. 10.1038/cr.2011.13 21243012 PMC3193440

[B22] Hu J. Chen Y. Huang Y. Su Y. (2020). Human umbilical cord mesenchymal stem cell-derived exosomes suppress dermal fibroblasts-myofibroblats transition via inhibiting the TGF-ß1/smad 2/3 signaling pathway. Exp. Mol. Pathology 115, 104468. 10.1016/j.yexmp.2020.104468 32445750

[B23] Joo H. S. Suh J. H. Lee H. J. Bang E. S. Lee J. M. (2020). Current knowledge and future perspectives on mesenchymal stem cell-derived exosomes as a new therapeutic agent. Int. J. Mol. Sci. 21 (3), E727. 10.3390/ijms21030727 PMC7036914 31979113

[B24] Korbecki J. Simińska D. Gąssowska-Dobrowolska M. Listos J. Gutowska I. Chlubek D. (2021). Chronic and cycling hypoxia: Drivers of cancer chronic inflammation through HIF-1 and NF-?b activation: A Review of the molecular mechanisms. Int. J. Mol. Sci. 22 (19), 10701. 10.3390/ijms221910701 34639040 PMC8509318

[B25] Lai G. Zhao R. Zhuang W. Hou Z. Yang Z. He P. (2022). BMSC-derived exosomal MiR-27a-3p and MiR-196b-5p regulate bone remodeling in ovariectomized rats. PeerJ 10, e13744. 10.7717/peerj.13744 36168439 PMC9509671

[B26] Lee C. Mitsialis S. A. Aslam M. Vitali S. H. Vergadi E. Konstantinou G. (2012). Exosomes mediate the cytoprotective action of mesenchymal stromal cells on hypoxia-induced pulmonary hypertension. Circulation 126 (22), 2601–2611. 10.1161/CIRCULATIONAHA.112.114173 23114789 PMC3979353

[B27] Lee I. Y. Lim J. M. Cho H. Kim E. Kim Y. Oh H.-K. (2019). MST1 negatively regulates TNFα-induced NF-?b signaling through modulating LUBAC activity. Mol. Cell. 73 (6), 1138–1149. e6, March 21. 10.1016/j.molcel.2019.01.022 30901564

[B28] Leibowitz S. M. Yan J. (2016). NF-ΚB pathways in the pathogenesis of multiple sclerosis and the therapeutic implications. Front. Mol. Neurosci. 9, 84. 10.3389/fnmol.2016.00084 27695399 PMC5023675

[B29] Li L. Yu R. Cai T. Chen Z. Lan M. Zou T. (2020). Effects of immune cells and cytokines on inflammation and immunosuppression in the tumor microenvironment. Int. Immunopharmacol. 88, 106939. 10.1016/j.intimp.2020.106939 33182039

[B30] Li P. Lv S. Jiang W. Si L. Liao B. Zhao G. (2022). Exosomes derived from umbilical cord mesenchymal stem cells protect cartilage and regulate the polarization of macrophages in osteoarthritis. Ann. Transl. Med. 10 (18), 976. 10.21037/atm-22-3912 36267713 PMC9577719

[B31] Liang X. Ding Y. Zhang Y. Tse H.-F. Lian Q. (2014). Paracrine mechanisms of mesenchymal stem cell-based Therapy: Current status and perspectives. Cell. Transplant. 23 (9), 1045–1059. 10.3727/096368913X667709 23676629

[B32] Liao W. Tang X. Li X. Li T. (2019). Therapeutic effect of human umbilical cord mesenchymal stem cells on tubal factor infertility using a chronic salpingitis murine model. Archives Gynecol. Obstetrics 300 (2), 421–429. 10.1007/s00404-019-05209-6 31190174

[B33] Lin Z. Wu Y. Xu Y. Li G. Li Z. Liu T. (2022). Mesenchymal stem cell-derived exosomes in cancer Therapy resistance: Recent advances and therapeutic potential. Mol. Cancer 21 (1), 179. 10.1186/s12943-022-01650-5 36100944 PMC9468526

[B34] Ling H. Luo L. Dai X. Chen H. (2022). Fallopian tubal infertility: The result of Chlamydia trachomatis-induced fallopian tubal fibrosis. Mol. Cell. Biochem. 477 (1), 205–212. 10.1007/s11010-021-04270-7 34652537

[B35] Liu M. Sun Y. Zhang Q. (2018). Emerging role of extracellular vesicles in bone remodeling. J. Dent. Res. 97 (8), 859–868. 10.1177/0022034518764411 29566346

[B36] Logozzi M. Mizzoni D. Di Raimo R. Fais S. (2020). Exosomes: A source for new and old biomarkers in cancer. Cancers 12 (9), E2566. 10.3390/cancers12092566 PMC7565506 32916840

[B37] Luo X. Xu J. Zhao R. Qin J. Wang X. Yan Y. (2022). The role of inactivated NF-?b in premature ovarian failure. Am. J. Pathology 192 (3), 468–483. 10.1016/j.ajpath.2021.12.005 34971586

[B38] Markov A. Thangavelu L. Aravindhan S. Zekiy A. O. Jarahian M. Chartrand M. S. (2021). Mesenchymal stem/stromal cells as a valuable source for the treatment of immune-mediated Disorders. Stem Cell. Res. Ther. 12 (1). 192. 10.1186/s13287-021-02265-1 PMC7971361 33736695

[B39] Mascarenhas M. N. Flaxman S. R. Boerma T. Vanderpoel S. Stevens G. A. (2012). National, regional, and global trends in infertility prevalence since 1990: A systematic analysis of 277 Health surveys. PLoS Med. 9 (12), e1001356. 10.1371/journal.pmed.1001356 23271957 PMC3525527

[B40] McKay T. B. Yeung V. Hutcheon A. E. K. Guo X. Zieske J. D. Ciolino J. B. (2021). Extracellular vesicles in the cornea: Insights from other tissues. Anal. Cell. Pathol. Amst., 9983900. 10.1155/2021/9983900 34336556 PMC8324376

[B41] Medzhitov R. (2008). Origin and physiological roles of inflammation. Nature 454 (7203), 428–435. 10.1038/nature07201 18650913

[B42] Meng F. Xu R. Wang S. Xu Z. Zhang C. Li Y. (2020). Human umbilical cord-derived mesenchymal stem cell Therapy in patients with COVID-19: A phase 1 clinical trial. Signal Transduct. Target. Ther. 5, 172. 10.1038/s41392-020-00286-5 32855385 PMC7450163

[B43] Mitchell J. P. Carmody R. J. (2018). NF-ΚB and the transcriptional control of inflammation. Int. Rev. Cell. Mol. Biol. 335, 41–84. 10.1016/bs.ircmb.2017.07.007 29305014

[B44] Murthy A. K. Li W. Ramsey K. H. (2018). Immunopathogenesis of chlamydial infections. Curr. Top. Microbiol. Immunol. 412, 183–215. 10.1007/82_2016_18 27370346 PMC6086136

[B45] Negi N. Griffin M. D. (2020). Effects of mesenchymal stromal cells on regulatory T cells: Current understanding and clinical relevance. Stem Cells Dayt. Ohio) 38 (5), 596–605. 10.1002/stem.3151 PMC7217190 31995249

[B46] Newman L. Rowley J. Vander Hoorn S. Wijesooriya N. S. Unemo M. Low N. (2015). Global estimates of the prevalence and incidence of four curable sexually transmitted infections in 2012 based on systematic Review and global reporting. PLoS ONE 10 (12), e0143304. 10.1371/journal.pone.0143304 26646541 PMC4672879

[B47] Ning Y. Cui Y. Li X. Cao X. Chen A. Xu C. (2018). Co-culture of ovarian cancer stem-like cells with macrophages induced SKOV3 cells stemness via IL-8/STAT3 signaling. Biomed. Pharmacother. = Biomedecine Pharmacother. 103, 262–271. 10.1016/j.biopha.2018.04.022 29656182

[B48] Pegtel D. M. Gould S. J. (2019). Exosomes, Annu. Rev. Biochem. 88 (1), 487–514. 10.1146/annurev-biochem-013118-111902 31220978

[B49] Pluchino S. Smith J. A. (2019). Explicating exosomes: Reclassifying the rising stars of intercellular communication. Cell. 177 (2), 225–227. 10.1016/j.cell.2019.03.020 30951665

[B50] Pouyanfard S. Meshgin N. Cruz L. S. Diggle K. Hashemi H. Pham T. V. (2021). Human induced pluripotent stem cell-derived macrophages ameliorate liver fibrosis. Stem Cells Dayt. Ohio) 39 (12), 1701–1717. 10.1002/stem.3449 34460131

[B51] Qian M. Wang S. Guo X. Wang J. Zhang Z. Qiu W. (2020). Hypoxic glioma-derived exosomes deliver MicroRNA-1246 to induce M2 macrophage polarization by targeting TERF2IP via the STAT3 and NF-?b pathways. Oncogene 39 (2). 10.1038/s41388-019-0996-y 31485019

[B52] Qin Y. Wang L. Gao Z. Chen G. Zhang C. (2016). Bone marrow stromal/stem cell-derived extracellular vesicles regulate osteoblast activity and differentiation *in vitro* and promote bone regeneration *in vivo* . Sci. Rep. 6, 21961. 10.1038/srep21961 26911789 PMC4766421

[B53] Qiu G. Zheng G. Ge M. Wang J. Huang R. Shu Q. (2019). Functional proteins of mesenchymal stem cell-derived extracellular vesicles. Stem Cell. Res. Ther. 10 (1), 359. 10.1186/s13287-019-1484-6 31779700 PMC6883709

[B54] Reis M. Willis G. R. Fernandez-Gonzalez A. Yeung V. Taglauer E. Magaletta M. (2021). Mesenchymal stromal cell-derived extracellular vesicles Restore thymic architecture and T cell function disrupted by neonatal hyperoxia. Front. Immunol. 12, 640595. 10.3389/fimmu.2021.640595 33936055 PMC8082426

[B55] Rong X. Liu J. Yao X. Jiang T. Wang Y. Xie F. (2019). Human bone marrow mesenchymal stem cells-derived exosomes alleviate liver fibrosis through the wnt/β-catenin pathway. Stem Cell. Res. Ther. 10 (1), 98. 10.1186/s13287-019-1204-2 30885249 PMC6421647

[B56] Sengupta V. Sengupta S. Lazo A. Woods P. Nolan A. Bremer N. (2020). Exosomes derived from bone marrow mesenchymal stem cells as treatment for severe COVID-19. Stem Cells Dev. 29 (12), 747–754. 10.1089/scd.2020.0080 32380908 PMC7310206

[B57] Seo Y. Kim H.-S. Hong I.-S. (2019). Stem cell-derived extracellular vesicles as immunomodulatory therapeutics. Stem Cells Int. 2019, 5126156. 10.1155/2019/5126156 30936922 PMC6413386

[B58] Shao L.-T. Luo L. Qiu J.-H. Deng D. Y. B. (2022). PTH (1-34) enhances the therapeutic effect of bone marrow mesenchymal stem cell-derived exosomes by inhibiting proinflammatory cytokines expression on OA chondrocyte repair *in vitro* . Arthritis Res. Ther. 24 (1), 96. 10.1186/s13075-022-02778-x 35488245 PMC9052609

[B59] Shao M. Xu Q. Wu Z. Chen Y. Shu Y. Cao X. 2020. Exosomes derived from human umbilical cord mesenchymal stem cells ameliorate IL-6-induced acute liver injury through MiR-455-3p. Stem Cell. Res. Ther. 11 (1). 10.1186/s13287-020-1550-0 PMC6979401 31973730

[B60] Shao R. Wang X. Wang W. Stener-Victorin E. Mallard C. Brännström M. (2012). From mice to women and back again: Causalities and clues for chlamydia-induced tubal ectopic pregnancy. Fertil. Steril. 98 (5), 1175–1185. 10.1016/j.fertnstert.2012.07.1113 22884019

[B61] Shapouri-Moghaddam A. Mohammadian S. Vazini H. Taghadosi M. Esmaeili S.-A. Mardani F. (2018). Macrophage plasticity, polarization, and function in Health and disease. J. Cell. Physiology 233 (9), 6425–6440. 10.1002/jcp.26429 PMC13482560 29319160

[B62] Shi Y. Wang Y. Li Q. Liu K. Hou J. Shao C. (2018). Immunoregulatory mechanisms of mesenchymal stem and stromal cells in inflammatory diseases. Nat. Rev. Nephrol. 14 (8), 493–507. 10.1038/s41581-018-0023-5 29895977

[B63] Simons M. Raposo G. (2009). Exosomes – vesicular carriers for intercellular communication. Curr. Opin. Cell. Biol. 21 (4), 575–581. 10.1016/j.ceb.2009.03.007 19442504

[B64] Sun S.-C. (2017). The non-canonical NF-?b pathway in immunity and inflammation. Nat. Rev. Immunol. 17 (9), 545–558. 10.1038/nri.2017.52 28580957 PMC5753586

[B65] Sun X. Luan Q. Qiu S. (2018). Valsartan prevents glycerol-induced acute kidney injury in male albino rats by downregulating TLR4 and NF-?b expression. Int. J. Biol. Macromol. 119, 565–571. 10.1016/j.ijbiomac.2018.07.149 30053391

[B66] Taglauer E. S. Fernandez-Gonzalez A. Willis G. R. Reis M. Yeung V. Liu X. (2022). Antenatal mesenchymal stromal cell extracellular vesicle Therapy prevents preeclamptic lung injury in mice. Am. J. Respir. Cell. Mol. Biol. 66 (1), 86–95. 10.1165/rcmb.2021-0307OC 34614384 PMC8803363

[B67] Tang L. Jiang Y. Zhu M. Chen L. Zhou X. Zhou C. (2020). Clinical study using mesenchymal stem cells for the treatment of patients with severe COVID-19. Front. Med. 14 (5), 664–673. 10.1007/s11684-020-0810-9 32761491 PMC7406954

[B68] Taniguchi K. Karin M. (2018). NF-ΚB, inflammation, immunity and cancer: Coming of age. Nat. Rev. Immunol. 18 (5), 309–324. 10.1038/nri.2017.142 29379212

[B69] Teng L. Maqsood M. Zhu M. Zhou Y. Kang M. Zhou J. (2022). Exosomes derived from human umbilical cord mesenchymal stem cells accelerate diabetic wound healing via promoting M2 macrophage polarization, angiogenesis, and collagen deposition. Int. J. Mol. Sci. 23 (18), 10421. 10.3390/ijms231810421 36142334 PMC9498995

[B70] Tsevat D. G. Wiesenfeld H. C. Parks C. Peipert J. F. (2017). Sexually transmitted diseases and infertility. Am. J. Obstetrics Gynecol. 216 (11–9). 10.1016/j.ajog.2016.08.008 PMC5193130 28007229

[B71] Ullah M. Liu D. D. Rai S. Razavi M. Concepcion W. Thakor A. S. (2020). Pulsed focused ultrasound enhances the therapeutic effect of mesenchymal stromal cell-derived extracellular vesicles in acute kidney injury. Stem Cell. Res. Ther. 11 (1), 398. 10.1186/s13287-020-01922-1 32928310 PMC7490886

[B72] Vizoso F. J. Eiro N. Cid S. Schneider J. Perez-Fernandez R. (2017). Mesenchymal stem cell secretome: Toward cell-free therapeutic strategies in regenerative medicine. Int. J. Mol. Sci. 18 (9), E1852. 10.3390/ijms18091852 PMC5618501 28841158

[B73] Webber J. Yeung V. Clayton A. (2015). Extracellular vesicles as modulators of the cancer microenvironment. Seminars Cell. & Dev. Biol. 40, 27–34. 10.1016/j.semcdb.2015.01.013 25662446

[B74] Weng Z. Zhang B. Wu C. Yu F. Han B. Li B. (2021). Therapeutic roles of mesenchymal stem cell-derived extracellular vesicles in cancer. J. Hematol. Oncol. 14 (1), 136. 10.1186/s13045-021-01141-y 34479611 PMC8414028

[B75] Willis G. R. Fernandez-Gonzalez A. Reis M. Yeung V. Liu X. Ericsson M. (2020). Mesenchymal stromal cell-derived small extracellular vesicles Restore lung architecture and improve exercise capacity in a model of neonatal hyperoxia-induced lung injury. J. Extracell. Vesicles 9 (1), 1790874. 10.1080/20013078.2020.1790874 32939235 PMC7480622

[B76] Willis G. R. Reis M. Gheinani A. H. Fernandez-Gonzalez A. Taglauer E. S. Yeung V. (2021). Extracellular vesicles protect the neonatal lung from hyperoxic injury through the epigenetic and transcriptomic reprogramming of myeloid cells. Am. J. Respir. Crit. Care Med. 204 (12), 1418–1432. 10.1164/rccm.202102-0329OC 34699335 PMC8865710

[B77] Xin L. Lin X. Zhou F. Li C. Wang X. Yu H. (2020). A scaffold laden with mesenchymal stem cell-derived exosomes for promoting endometrium regeneration and fertility restoration through macrophage immunomodulation. Acta Biomater. 113, 252–266. 10.1016/j.actbio.2020.06.029 32574858

[B78] Xu J.-J. Wang G. Luo X. Wang L.-J. Bao Y. Yang X. (2019). Role of nuclear factor-?b pathway in the transition of mouse secondary follicles to antral follicles. J. Cell. Physiology 234 (12), 22565–22580. 10.1002/jcp.28822 31102283

[B79] Yang C. Lei L. Collins J. W. M. Briones M. Ma L. Sturdevant G. L. (2021). Chlamydia evasion of neutrophil host defense results in NLRP3 dependent myeloid-mediated sterile inflammation through the purinergic P2X7 receptor. Nat. Commun. 12 (1), 5454. 10.1038/s41467-021-25749-3 34526512 PMC8443728

[B80] Yatim N. Jusforgues-Saklani H. Orozco S. Schulz O. Barreira da Silva R. Reis e Sousa C. (2015). RIPK1 and NF-?b signaling in dying cells determines cross-priming of CD8^+^ T cells. Sci. (New York, N.Y.) 350 (6258), 328–334. 10.1126/science.aad0395 PMC4651449 26405229

[B81] Yeung V. Willis G. R. Taglauer E. (2019). Stem cell-based Therapy for lung disease. New York: Springer.

[B82] Yeung Vincent Zhang T. C. Yuan L. Parekh M. Cortinas J. A. Delavogia E. (2022). Extracellular vesicles secreted by corneal myofibroblasts promote corneal epithelial cell migration. Int. J. Mol. Sci. 23 (6), 3136. 10.3390/ijms23063136 35328555 PMC8951135

[B83] Yin S. Ji C. Wu P. Jin C. Qian H. (2019). Human umbilical cord mesenchymal stem cells and exosomes: Bioactive ways of tissue injury repair. Am. J. Transl. Res. 11 (3), 1230–1240. 30972158 PMC6456565

[B84] Yonke N. Aragón M. Phillips J. K. (2022). Chlamydial and gonococcal infections: Screening, diagnosis, and treatment. Am. Fam. Physician 105 (4), 388–396. 35426632

[B85] Yu H. Lin L. Zhang Z. Zhang H. Hu H. (2020). Targeting NF-?b pathway for the Therapy of diseases: Mechanism and clinical study. Signal Transduct. Target. Ther. 5 (1). 209. 10.1038/s41392-020-00312-6 32958760 PMC7506548

[B86] Yuan X. Yuan W. Ding L. Shi M. Luo L. Wan Y. (2021). Cell-adaptable dynamic hydrogel reinforced with stem cells improves the functional repair of spinal cord injury by alleviating neuroinflammation. Biomaterials 279, 121190. 10.1016/j.biomaterials.2021.121190 34736145

[B87] Zhang M. Johnson-Stephenson T. K. Wang W. Wang Y. Li J. Li L. (2022). Mesenchymal stem cell-derived exosome-educated macrophages alleviate systemic lupus erythematosus by promoting efferocytosis and recruitment of IL-17+ regulatory T cell. Stem Cell. Res. Ther. 13 (1), 484. 10.1186/s13287-022-03174-7 36153633 PMC9509559

[B88] Zhang M. Liu Z. Z. Aoshima K. Cai W. L. Sun H. Xu T. (2022). CECR2 drives breast cancer metastasis by promoting NF-?b signaling and macrophage-mediated immune suppression. Sci. Transl. Med. 14 (630), eabf5473. 10.1126/scitranslmed.abf5473 35108062 PMC9003667

