# Supplementary material for: Human umbilical cord mesenchymal stem cells derived extracellular vesicles alleviate salpingitis by promoting M1–to–M2 transformation
Source: Front Physiol. 2023 Feb 16;14:1131701. doi: 10.3389/fphys.2023.1131701 (PMC9977816; doi:10.3389/fphys.2023.1131701)
Supplement: Supplementary file 1 [file DataSheet1.docx]

Supplementary Material

Exosomes Derived from Human Umbilical Cord Mesenchymal Stem Cells Alleviate Salpingitis by Promoting M1–to–M2 Transformation

Changlin Zhang^1†^, Wei Liao^1†^, Weizhao Li^1†^, Mengxiong Li^1^, Xiaoyu Xu^1^, Haohui Sun^2^, Yaohua Xue^3^, Lixiang Liu^1^, Jiehong Qiu^1^, Chi Zhang^1^, Xunzhi Zhang^4^, Juntong Ye^1^, Jingran Du^1^, David YB Deng^1^*, Wuguo Deng^2^*, Tian Li^1^*

*** Correspondence:** Tian Li: litian@sysush.com

# Supplementary Figures and Tables

## Supplementary Figure





**Supplementary Figure 1.** Modeling process in animal experiments. The mice were injected medroxyprogesterone first, and then transvaginal injected MoPn Chlamydia. After that, the mice were injected with hucMSC-EXO or DPBS or DMEM media, and then sacrificed for test or fertilized to see the pregnancy rate.

## Supplementary table

| **Supplementary Table 1.** Primer sequences used for quantitative RT-PCR | | | |
| --- | --- | --- | --- |
| Genes | Primer sequence (5′–3′) | | Amplicon size (bp) |
| GAPDH | F:CATCACTGCCACCCAGAAGACTG | R:ATGCCAGTGAGCTTCCCGTTCAG | 23 |
| F4/80 | F:CGTGTTGTTGGTGGCACTGTGA | R:CCACATCAGTGTTCCAGGAGAC | 133 |
| iNOS | F:GAGACAGGGAAGTCTGAAGCAC | R:CCAGCAGTAGTTGCTCCTCTTC | 127 |
| CD206 | F:GTTCACCTGGAGTGATGGTTCTC | R:AGGACATGCCAGGGTCACCTTT | 116 |
| TNF-α | F:CCCTCACACTCAGATCATCTTCT | R:GCTACGACGTGGGCTACAG | 139 |
| IL-1β | F:TGGACCTTCCAGGATGAGGACA | R:GTTCATCTCGGAGCCTGTAGTG | 148 |
| IL-10 | F:CAGTACAGCCGGGAAGACAA | R:TGGCAACCCAAGTAACCCTTA | 130 |
